# A rare posterior mediastinal mass: chordoma

**DOI:** 10.1093/jscr/rjae299

**Published:** 2024-05-07

**Authors:** Lily Frances Stocking Willatt, Martina Henrietta Wollheim, Jonas Peter Ehrsam, Beata Bode-Lesniewska, Christoph Woernle, Othmar Schoeb, Ilhan Inci

**Affiliations:** University of Nicosia, Makedonitissas Avenue, CY-2417, Nicosia, Cyprus; Surgery, Klinik Hirslanden, Witellikerstrasse 40, 8032, Zürich, Switzerland; University of Nicosia, Makedonitissas Avenue, CY-2417, Nicosia, Cyprus; Surgery, Klinik Hirslanden, Witellikerstrasse 40, 8032, Zürich, Switzerland; Surgery, Klinik Hirslanden, Witellikerstrasse 40, 8032, Zürich, Switzerland; Pathology Institute Enge, Hardturmstrasse 133, 8005, Zürich, Switzerland; University of Zürich, Rämistrasse 71, 8006, Zürich, Switzerland; Neurosurgery, Klinik Hirslanden, Witellikerstrasse 40, 8032, Zürich, Switzerland; Surgery, Klinik Hirslanden, Witellikerstrasse 40, 8032, Zürich, Switzerland; University of Zürich, Rämistrasse 71, 8006, Zürich, Switzerland; Surgery, Klinik Hirslanden, Witellikerstrasse 40, 8032, Zürich, Switzerland; University of Zürich, Rämistrasse 71, 8006, Zürich, Switzerland

**Keywords:** chordoma, posterior mediastinal mass, paravertebral mass, Di Vinci, case report

## Abstract

A 72-year-old female presented with 2 years of pro-gradient pain in the upper thoracic spine radiating to the left arm and leg. MRI revealed a 2.7 × 2.0 × 12 cm paravertebral mass at T2/T3, extending into the foraminal and epidural nerves with extensive dural sac contact in the left hemithorax. The removed tumour was surprisingly soft for a schwannoma or chordoma. However, after the surgery, histopathology revealed the presence of brachyury protein (T-box transcription factor T), which is characteristic of a chordoma. While chordomas are extremely rare, it is important that they are kept in mind for the differential diagnosis of a posterior mediastinal mass. Successful treatment can only be achieved through a complete en bloc resection. This can often be complex due to their location along the spine. This case report aims to highlight the features and treatment of this rare disease.

## Introduction

Chordomas are rare, malignant, slow-growing, and locally destructive tumours derived from notochord cells anywhere along the spine [1]. They have an incidence of 8 per 10 million people per year [2]. Their 5-year survival rate is 50% [3]. They often cause back pain. Surgical en-bloc resection, with negative margins, is the only curable treatment for this disease. To date, knowledge of these rare tumours is limited to case reports. Thus, we aim to highlight this rare disease and its management.

## Case presentation

**Figure 1:**
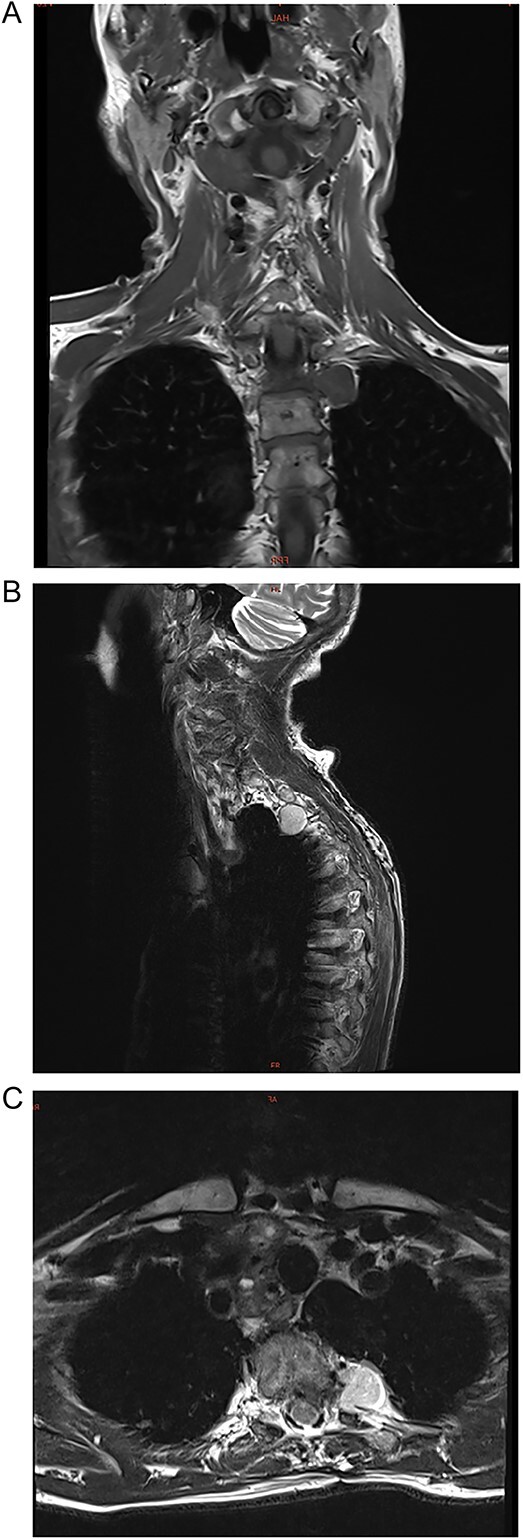
(A–C) Coronal, sagittal, and axial images of a 2.7 × 2.0 × 1.2 cm para-vertebral mass in the left hemithorax at the thoracic vertebrae T2/T3 level.

A 72-year-old female presented to neurology with a 2-year history of progressive pain in the upper thoracic spine. The pain radiated to her left arm and leg. She was non-responsive to pain relief but had no functional deficits. Her only relevant past medical history was multi-segmental degenerative changes in the lumbar and cervical spine. She took no known medications. A physical examination revealed pain in the upper thoracic spine with no other findings. Her routine blood examination was unremarkable. Chest magnetic resonance imaging (MRI) revealed a 2.7 × 2.0 × 1.2 cm para-vertebral mass in the left hemithorax at the thoracic vertebrae T2/T3 (Fig. 1). It extended into the foramina and had extensive dural sac contact. It had smooth contours with no bony destruction.

Differential diagnosis included neurogenic tumours, including nerve sheath tumours, such as schwannomas and neurofibromas, parasympathetic ganglion tumours, and sympathetic chain tumours.

It also included non-neurogenic tumours such as chordoma, chondrosarcoma, and Ewing’s sarcoma.

An intra-operative biopsy or surgical excision was the only way to make a diagnosis.

**Figure 2:**
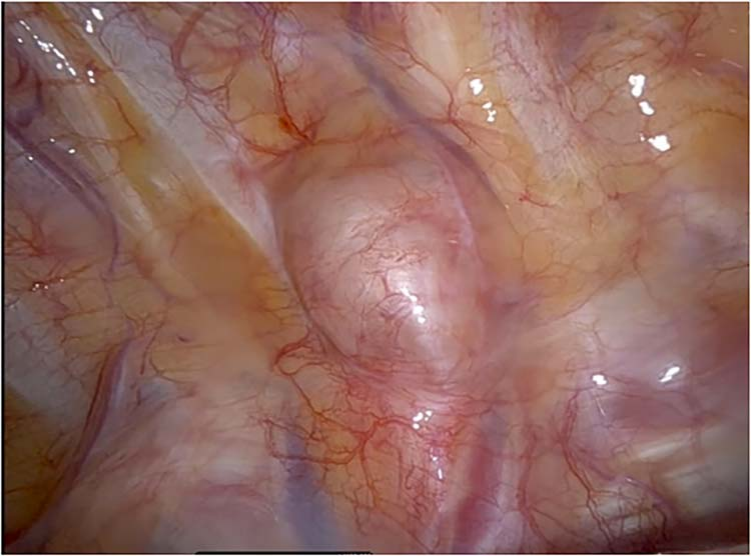
Intra-operative photo showing the lesion *in situ*, located in the 3rd intercostal space, close to the vertebra. It is covered with mediastinal pleura and slightly vascularized.

The patient was referred to our thoracic surgery department, where we performed robot-assisted (Di Vinci Xi) para-vertebral en-bloc tumour resection (Fig. 2). A neurosurgeon was present for the duration of the surgery. A complete resection was successful without disturbance of the dural sac, preventing infection of the cerebrospinal fluid. The operation time was 65 min with a blood loss of <20 ml.

**Figure 3:**
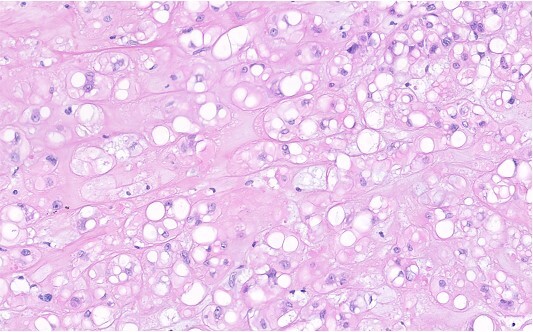
H&E stain of chordoma tissue with chondromyxoid background. The tumour cells grow in nests and cords. They contain middle sized, moderately pleomorphic nuclei with small nucleoli and broad, and often vacuolated eosinophilic cytoplasm, all features typical for chordoma tissue.

**Figure 4:**
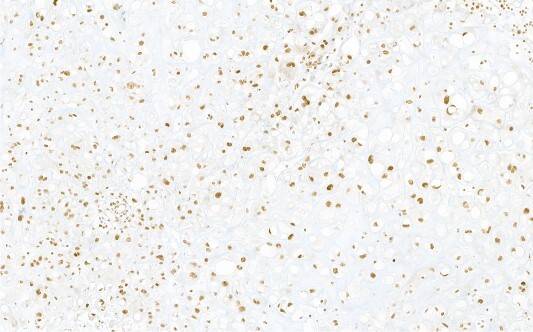
Immunohistochemical reaction for chordoma-specific antibody brachyury, demonstrating typical nuclear expression.

Histology showed a soft, lobular structure with a myxoid ground substance (Fig. 3). It had a cell population of epithelial cells and eosinophilic cytoplasmic borders. The immune profile (Fig. 4) showed co-expression of pan-cytokeratins, epithelial antigen, S100-protein positivity, and brachyury protein (T-box transcription factor T) with clear nuclear expression, which is characteristic of a chordoma.

Patient follow-up was uncomplicated. The patient was extubated in the operating theatre, and the chest tube was removed on the first postoperative day. She was discharged on Day 3 with analgesia and wound control. On a 3-week follow-up, she had minimal pain. Six months and one year of follow-up MRIs showed no tumour recurrence.

## Discussion

Chordomas are rare, locally invasive, and malignant tumours derived from notochord cells of the spine. The notochord is the embryonic midline structure, which provides signals during development [4]. A cancer causes control study of 400 cases in the US found that 32% of the chordomas were cranial, 32.8% were spinal, and 29.2% were sacral [2]. Cranial chordomas tend to produce symptoms, such as headache, neck pain, and cranial nerve palsies. Spine and sacral tumours tend to cause chronic back pain and urinary or bowel dysfunction [3]. They are most common between the ages of 40–50 years. In patients younger than 20, they are usually skull-based chordomas [3]. They tend to metastasize to the lung, liver, and bone [5].

An MRI is performed to make a differential diagnosis. Chordomas will appear isointense relative to the muscle on T1-weighted imaging versus hyper-intense on T2-weighted imaging due to their myxoid matrix. Usually, they will show a lobular appearance. Computed tomography (CT) can also be performed and will show an expansile lobulated mass with lytic bone destruction and sometimes calcification due to entrapped bone [6].

It is important to note that 95% of posterior mediastinal masses are neurogenic. This includes nerve sheath tumours, including schwannomas, parasympathetic ganglion tumours, and sympathetic chain tumours [7]. The other 5% is made up of non-neurogenic tumours, such as chordoma, chondrosarcoma, Ewing’s sarcoma, oesophageal neoplasms, lymphoma, and invasive thymoma.

One case report describes a 60-year-old man with a left paraspinal mass from T7 to T9 assumed to be a schwannoma. An attempted resection had to be aborted due to excess bleeding due to its proximity to the intercostal arteries and aorta. A biopsy was successfully completed and revealed that the tumour was actually a chordoma. On CT, the mass showed obliteration of the plane between the spine and tumour, more characteristic of a chordoma [8]. This case highlights the importance of chordomas in the differential diagnosis, despite their rarity.

Benign masses that should be considered in the differential diagnosis of a chordoma are ecchordosis physaliphora (EP) and benign notchordal cell tumours (BNCT) [9]. Both are small, asymptomatic entities, with well-defined borders. They do not have a lobular structure, lack necrosis, and are non-invasive, unlike chordomas. Chordomas also show contrast enhancement on MRI, unlike EP or BNCT [6].

The WHO defines three types of chordomas: conventional, dedifferentiated, and poorly differentiated chordomas [6]. Histologically, chordomas have a lobulated appearance with fibrous bands. The lobules contain large epithelial cells, an eosopnophilic cytoplasm, and a myxoid matrix. Necrosis is usually present and can be severe. Dedifferentiated chordomas tend to have a sarcoma component. The poorly differentiated type is usually found in younger patients and is characterized by SMARCB1/INI loss [10].

On immunohistochemistry, they express various keratins, epithelial membrane antigen (EMA), S100 protein, and brachyury (T-box transcription factor T) [6]. Brachyury is also expressed in the foetal notochord, but not in many other types of neoplasm, making it its most characteristic feature [11].

Surgical en-bloc resection with negative microscopic wide margins is the sole treatment for chordomas [12]. Surgical techniques differ depending on the location of the mass. Removal can be challenging due to the nature of its location, and therefore radiotherapy is often recommended.

## Conflict of interest statement

None declared.

## Funding

None declared.
